# Impact of particle size and associated minerals on rare earth desorption and incorporation mechanisms in a South American ion-adsorption clay

**DOI:** 10.1038/s41598-024-67134-2

**Published:** 2024-07-13

**Authors:** Lingyang Ding, Gisele Azimi

**Affiliations:** Laboratory for Strategic Materials, Department of Chemical Engineering and Applied Chemistry, University of Toronto, 200 College Street, Toronto, ON M5S 3E5 Canada

**Keywords:** Ion-adsorption clay, Rare earth element, Desorption, Physiosorbed, Chemisorbed, Particle size, South America, Chemical engineering, Chemical bonding

## Abstract

This research delves into the intricate nexus of particle size, mineralogical composition, surface attributes, elemental mapping, and rare earth element (REE) adsorption mechanisms within an ion-adsorption clay sample from South America. The investigation entails the fractionation of the ion-adsorption clay into three size categories: S1 (< 0.25 mm), S2 (0.25–0.5 mm), and S3 (0.5–2 mm). Each fraction undergoes meticulous characterization to unveil its elemental composition, mineralogical composition, surface area, morphological characteristics, elemental mapping, and the mechanisms governing REE incorporation. The results indicate that S1 has 31% physiosorbed, 8% chemisorbed, and 61% mineralized REEs, while S2 has 40% physiosorbed, 5% chemisorbed, and 55% mineralized REEs, and S3 has 24% physiosorbed, 5% chemisorbed, and 71% mineralized REEs. The physisorbed REEs are attributed to the presence of kaolinite, conducive to mostly physisorption. In terms of grain size and REE content/type relationship, the results show that REE content decreases with increasing grain size; however, there is not a clear trend in terms of REE occurrence modes with grain size. Heavy rare earth elements (HREEs) are discernibly favored in adsorption over light rare earth elements (LREEs). This preference is underpinned by the weathering processes that led to the formation of ion-adsorption clay, which facilitated the transport and accumulation of HREEs. Notably, the ion-adsorption clay encompasses a substantial content of mineralized REEs, necessitating more demanding extraction methodologies, such as acid baking followed by water leaching if complete extraction of all REEs is desired. Among the desorbable REEs, physisorption dominates, encompassing over 80% of the total. Chemisorbed REEs exhibit versatility in association with various minerals, encompassing kaolinite, quartz, and goethite. In essence, this study unveils the intricate interplay between particle dimensions, mineralogical constitution, surface attributes, and REE adsorption modes within this ion-adsorption clay sample. The ion-adsorption clay in this study contains a significant portion of mineralized REEs that cannot be extracted using the mild conditions typically employed for the desorption process. Additionally, the REE concentration in this ion-adsorption clay is notably higher than the average found in clay deposits worldwide, reaching levels comparable to those of regolith deposits in China, which are a major global source of REEs. This remarkable concentration of REEs, along with the unique modes of their occurrence in this deposit, presents a significant interest to the scientific community.

## Introduction

Rare earth elements (REEs) encompass a set of 17 elements, inclusive of the lanthanide series, along with yttrium (Y) and scandium (Sc). These elements possess distinct physicochemical characteristics that render them indispensable components of cutting-edge green technologies. Notably, they play a pivotal role in the creation of permanent magnets utilized in renewable energy applications like wind turbines and the motors of hybrid and electric vehicles. These technologies are of paramount importance for the global transition towards sustainable, low-carbon economies, particularly in light of the escalating demand for wind turbines and electric vehicles, which, in turn, fuels the need for REEs such as neodymium iron boron (NdFeB) magnets^1^.

The classification of REEs divides them into two distinct groups: light REEs (LREEs) and heavy REEs (HREEs). LREEs encompass elements with unpaired electrons in the 4f. orbitals, spanning from lanthanum (La) to gadolinium (Gd). On the other hand, HREEs encompass terbium (Tb) through lutetium (Lu). REEs are found in more than 200 types of minerals, but their main commercial sources are as follows ^2^: (1) Phosphates in the form of xenotime, monazite, and rhabdophane; (2) Carbonates in the form of bastnasite; and (3) Ion-adsorption clays.

The ion-adsorption clays contain around 0.05–0.5 wt% REO. REEs are adsorbed onto the surface of aluminosilicate minerals like kaolinite, illite, and smectite. Ion-adsorption clay deposits typically form due to the in-situ weathering of rocks containing REE bearing minerals, such as granite or other igneous rocks. This weathering results in the creation of aluminosilicate clays that adsorb solubilized REE ions. These deposits are most common in subtropical climates and are found in regions like southern China (latitudes 24–26°N) and South America, including Brazil and Chile.

Among the three types of resources mentioned, carbonates and phosphates pose challenges in terms of solubilizing REEs due to their resistance to dissolution. To make REEs soluble from monazite/xenotime concentrates, rigorous conditions are needed, such as treatment with 98 wt% H_2_SO_4_ or 70 wt% NaOH. Similarly, bastnasite necessitates concentrated H_2_SO_4_ or HCl for effective leaching. Conversely, ion-adsorption clays offer distinct advantages. They are relatively easy to mine and process, and they typically contain very low levels of radioactive elements. These clays are often extracted using open-pit methods and do not require beneficiation, resulting in reduced capital and operating costs as well as a smaller environmental footprint. The leaching process for ion-adsorption clays involves the use of mono- or divalent salt solutions at ambient temperatures to desorb REEs from the clay's surface. These advantages help offset the lower grade of ionic clays compared with other sources ^3^.

Previous studies have extensively examined the speciation and distribution of REEs across a wide range of pH and ionic strength conditions.^4–9^ These investigations have revealed that most of the REEs adsorbed on the surface result from reversible cation-exchange reactions. These reactions take place at the permanent negative charge sites present on the clay surface; a process referred to as physisorption. Additionally, REEs can exist as hydrolyzed "clay-O-REE" species due to permanent complexation reactions occurring at the amphoteric surface hydroxyl groups. This type of interaction is known as chemisorption ^2,10,11^.

Physisorption (Reaction 1) of REEs on ion-adsorption clay increases with increasing pH due to enhanced hydrolysis of REEs. Thus, physisorbed REEs can be released using sufficient lixiviant without adjusting the pH. On the other hand, chemisorption (Reaction 2), an exothermic process, primarily occurs at higher pH levels. Effective desorption of chemisorbed REEs requires lowering the pH and possibly the temperature. It is well-established that chemisorption of REEs, such as their binding to iron oxides via inner-sphere complex formation, is more effective at higher pH levels. Typically, both physisorption and chemisorption of cationic species like REE^3+^ are more pronounced at higher pH, while lower pH conditions are more conducive to the adsorption of anionic species, such as phosphate ions (PO_4_^3−^)^12,13^.

1 $$[IC{]}_{(s)}^{3-}+{REE}_{(aq)}^{3+} \rightleftharpoons [ICREE{]}_{(s)}$$

2 $$\left[IC\right]-(OH{)}_{3(s)}+{REE}_{(aq)}^{3+}+3{H}_{2}{O}_{(l)} \rightleftharpoons [IC-{O}_{3}REE{]}_{(s)}+ 3{H}_{3}{O}_{(aq)}^{+}$$

where IC represents the ion-adsorption clay samples.

The primary approach for extracting REEs from ionic clays involves their leaching using inorganic salt solutions containing mono- or divalent cations. Commonly used solutions include (NH_4_)_2_SO_4_, Na_2_SO_4_, MgSO_4_, NH_4_Cl, and NaCl. This leaching process results in the desorption of physisorbed REEs, where these REEs are effectively replaced by the cations present in the leaching salt. As a result, the REEs transition into the solution in the form of soluble sulfates or chlorides (as seen in reactions 3–5).

Following the leaching stage, the REEs can be separated from the solution using various methods, such as solvent extraction, ion exchange using resins, selective precipitation using substances like oxalic acid or ammonium bicarbonate. These separated REEs can then be further processed into rare earth oxides (REOs) through a roasting step carried out at approximately 900 °C.

In recent years, there has been significant development in new separation technologies for REEs. These innovations include electrodialysis^14^, free flow electrophoresis (FFE), rapid solvent extraction (SX), molecular recognition technology (MRT), continuous ion chromatography, micro-fluidics ^15^, and membrane-supported solvent extraction^16^.

NH_4_Cl and NaCl:

3 $${[ICREE]}_{(s)}+3MC{l}_{(aq)}\rightleftharpoons [\text{IC}{M}_{3}{]}_{\left(s\right)}+REE{Cl}_{3(aq)}$$

(NH_4_)_2_SO_4_ and Na_2_SO_4_:

4 $${2[ICREE]}_{(s)}+3{M}_{2}{SO}_{4(aq)}\rightleftharpoons 2[\text{IC}{M}_{3}{]}_{\left(s\right)}+{REE}_{2}{(SO}_{4}{)}_{3(aq)}$$

MgSO_4_:

5 $${2[ICREE]}_{(s)}+3Mg{SO}_{4(aq)}\rightleftharpoons [{\text{IC}}_{2}{Mg}_{3}{]}_{\left(s\right)}+{REE}_{2}{(SO}_{4}{)}_{3(aq)}$$

In contrast to physiosorbed REEs, which can be relatively easily leached, chemisorbed REEs require more severe conditions, particularly a lower pH (around 3), to reverse the hydrolysis reaction (2). The prevailing forms of adsorption (physisorption vs. chemisorption), the varying binding affinities for different elements, and the maximum adsorption capacity of ion-adsorption clays have been the subject of prior research. A few studies have investigated the effect of the particle size on the desorption efficiency of ion-adsorption clay.^17–19^. Chen et al. focused on the analysis of clay mineral types, content, soil particle size, pH value, leaching solution concentration, and leaching rate in ion-absorbed rare earth ores. The study utilized a combination of regional rare earth mine soil surveys, in situ leaching profile monitoring, and indoor simulated leaching experiments. Particle size analysis was performed using wet sieving and laser particle size analysis to categorize the soils based on grain size. X-ray diffraction (XRD) was employed to identify and quantify clay minerals, followed by Rietveld analysis for precise measurement of mineral phases. The study found both unimodal and bimodal patterns in particle size distribution, with variations significantly affecting the leaching and adsorption behaviors of REEs. Key clay minerals identified included kaolinite, illite, chlorite, and vermiculite. Lower pH values and higher leaching solution concentrations favored the formation of distinct kaolinite horizons, enhancing the leaching process. Simulated leaching experiments helped in understanding the impact of different leaching parameters on REE recovery and clay mineral transformations. The study suggests that optimizing grain size distribution and understanding the clay mineralogy are crucial for improving the efficiency of REE recovery through leaching processes^17^.

Eliott et al. explored the presence and distribution of REEs in Georgia kaolin deposits, focusing particularly on the heavy-mineral fractions of these deposits which include minerals like zircon, monazite, and xenotime. Sample processing involved crushing and dispersion of raw kaolin ore, followed by sedimentation to segregate coarse gangue minerals. Heavy liquid separation involved using lithium metatungstate to separate heavy from light mineral fractions based on density. X-ray diffractometry and scanning electron microscopy (SEM) were used to identify and quantify mineral compositions. The study found significant enrichment of heavy rare-earth elements (HREEs) in the heavy-mineral subfractions of the kaolin deposits, with concentrations considerably higher than those in the upper continental crust. The REE distribution is heavily influenced by the mineral composition, particularly the presence of zircon, which despite its low quantity in the deposits, contributes significantly to the REE content due to its high REE-bearing capacity. The findings indicate that the REE content in these heavy-mineral fractions could be economically viable for mining, suggesting a potential domestic source of REEs, particularly the HREEs^19^.

Li and Zhou focused on understanding the geochemical and mineralogical processes that contribute to the concentration of HREEs in regolith-hosted deposits. Particle size distribution was analyzed using laser diffraction to determine the proportions of various particle size fractions. Scanning electron microscopy (SEM) and high-resolution transmission electron microscopy (HRTEM) were employed to observe the micro- to nano-scale structures and associations of clay minerals. X-ray Diffraction (XRD) was used to identify and quantify clay minerals, with particular attention to the transformation of halloysite to kaolinite. Fourier Transform Infrared Spectroscopy (FTIR) provided insights into the chemical bonds and mineral transformations within the clay particles. Specific surface area, pore volume, and cation exchange capacity measurements were conducted to understand the physical properties of clay fractions that might influence REE adsorption. The study highlights significant transformations of clay minerals within the soil profile, particularly the transformation of halloysite into more crystalline kaolinite forms as part of the weathering process. This transformation impacts the physicochemical properties of the clays, influencing their ability to adsorb REEs. Higher adsorption capacities for REEs were associated with halloysite-rich clays in the deeper regolith, which possess greater surface area and porosity compared to kaolinite-dominant clays in shallower soils. The conversion of muscovite and feldspars during weathering also contributed to the clay mineralogy and associated REE dynamics. This study provides a detailed geochemical and mineralogical framework for understanding the concentration of HREEs in regolith-hosted deposits, with significant implications for mineral exploration and extraction technologies^18^.

The current study focuses on classifying an ion-adsorption clay by sieving, which leads to differences in the amount of kaolinite among various particle size fractions. In industrial practice, sieving is performed to separate the larger particle that do not contribute to the desorption process and the result of this study identifies the cut off threshold for particle size. Therefore, the outcomes observed are primarily driven by changes in mineral composition associated with different particle size, rather than by the size of the particles themselves. Understanding how mineral composition varies with particle size in ion-adsorption clay deposits is crucial for optimizing extraction processes for REEs. This research is important because it provides insights into how different mineral compositions, which are influenced by particle size, affect the desorption mechanisms of REEs. By identifying these relationships, the study can help improve the efficiency and effectiveness of REE extraction, which is vital for numerous technological and industrial applications.

The current study introduces novel aspects and key differences compared with the previously reviewed studies on similar topics. Here are the main points of novelty and significance in this research:

1. Novelty in Mineral Composition and Particle Size Analysis:
  This research uniquely focuses on the effects of particle size and associated mineral compositions on the desorption and incorporation mechanisms of REEs in an ion-adsorption clay from South America. This focus is distinct because it examines the direct implications of varying particle sizes, which was not extensively explored in the comparative studies.
  Unlike the previous studies that mainly examined the general mineralogical properties and chemical behaviors of REEs in clays, this study delves into how specific particle sizes and their associated minerals influence REE desorption behaviors, providing a deeper understanding of the process dynamics.
2. Introduction of Chemisorbed REEs in Desorption Studies:
  This study highlights the presence and significance of chemisorbed REEs across different particle sizes, which is an aspect not thoroughly investigated in the other papers. This inclusion offers a more comprehensive view of the adsorption mechanisms that could affect industrial extraction processes.
3. Significance and Implications:
  The detailed analysis of the relationship between particle sizes, mineral composition, and REE adsorption forms provides critical insights that are pivotal for optimizing extraction processes. This is particularly important for industrial applications where understanding the nuances of mineral composition can lead to more efficient and cost-effective REE recovery strategies.
  The findings that heavy REEs (HREEs) are preferentially adsorbed and are more prevalent in finer particles are particularly significant. This could influence future exploration and processing strategies, emphasizing finer fractions that might yield higher HREE concentrations.
4. Technological and Methodological Advancements:
  The current methodological approach, using detailed fractionation and characterization of each particle size, provides a replicable model for future studies aiming to optimize REE extraction from ion-adsorption clays. In contrast to previous studies, Chen et al. focused broadly on grain size and clay mineral content affecting leaching behaviors without specific attention to the detailed mechanisms of REE desorption^17^. Elliot et al. explored REE distributions in Georgia kaolin deposits but did not delve into the specifics of how particle size influences mineral associations and desorption behaviors^19^. Li and Zhou’s study was more focused on geochemical and mineralogical processes in regolith-hosted deposits, with less emphasis on the practical implications of particle size on desorption efficiency^18^.
  The current research fills these gaps by linking particle size and mineral composition directly to REE adsorption and desorption mechanisms, providing a new layer of understanding that is crucial for both academic research and practical applications in mining and materials engineering. This makes the current study not only novel but also a significant contribution to the field of rare earth element recovery from ion-adsorption clays.
  In this research, an ion-adsorption clay sourced from South America, which exhibits unique properties of containing comparatively high REE content that are in the form of physisorbed, chemisorbed, and mineralized REEs was investigated. A sample of this ion-adsorption clay was separated into three distinct particle size ranges: S1 (< 0.25 mm), S2 (0.25–0.5 mm), and S3 (0.5–2 mm). The reason behind these particle size ranges was the suggestion from our industrial partner. The goal was to sieve a mixture of sand, slit, and clay and determine the contribution of each size fraction to total desorption efficiency and REE occurrences modes. Using this technique, the cut off value of the particle size could be determined.
  The objective was to explore how variations in particle size influence the mineral composition, the content of both physiosorbed and chemisorbed REEs, along with desorption efficiency. Desorption experiments were carried out using 0.15 mol/L (NH_4_)_2_SO_4_ at a liquid-to-solid (L/S) ratio of 3 mL/g and pH 3, which were determined as the optimal conditions in our previous study to desorb both physisorbed and chemisorbed REEs^11^. For determining the percentage of each REE incorporation mechanism (physiosorbed and chemisorbed) within the as-is ion-adsorption clay and its three size fractions (S1–S3), two types of experiments were performed, one in the presence of 0.15 mol/L (NH_4_)_2_SO_4_ and one without (NH_4_)_2_SO_4_. The slurry containing the clay and lixiviant in both tests was subjected to sulfuric acid addition and the pH of the slurry was decreased to 3. The same experiments were repeated at the native pH (~ 5) without adding sulfuric acid. By comparing the desorption efficiencies at the two conditions, contributions from the different REEs (physiosorbed and chemisorbed) were calculated. More details are provided in the Experimental section.
  To gain a comprehensive understanding of each size fraction's properties, surface area measurements were conducted employing Brunauer–Emmett–Teller (BET) surface area analysis. The aim was to explore whether there exists a correlation between surface area and REE content. Additionally, an electron probe microanalyzer (EPMA) was employed to assess particle morphology and conduct elemental mapping within each size fraction. These results offer insights into the physical characteristics and elemental distribution within the particles.
  It is important to note that the aim of this study is to classify the ion-adsorption clay by sieving, resulting in variations in the kaolinite content across different particle size fractions. Consequently, the observed effects are attributed to variations in mineral composition linked to particle size, rather than particle size directly influencing the outcomes.
  The outcomes of this study contribute to a more thorough understanding of the relationship between particle size, surface area, mineral composition, morphology, REE distribution, and the underlying incorporation forms within the ion-adsorption clay samples. Importantly, this clay differs significantly from most previously reported ion-adsorption clay samples, which predominantly contain adsorbed REEs in the form of physiosorbed or chemisorbed REEs^2,20^, whereas, the ion-adsorption clay in this study has significant portion of REEs as mineralized REEs that cannot be extracted under the mild conditions used for the desorption process. The REE concentration of this ion-adsorption clay is considerably higher than the average of clay deposits worldwide, indeed it is as high as regolith deposits in China that currently dominate the world supply of REEs^21^. This exceptional REE concentration makes the clay sample described in the manuscript, and most importantly, the modes of occurrence of REEs in this deposit very interesting to the scientific community.
  The findings of this study provide valuable insights into how mineral compositions differ in different particle sizes, and their joint impacts on REE adsorption and the associated desorption forms.

## Results and discussion

### Composition, mineralogy, and surface area of the ion-adsorption clay and sieved fractions

Table 1 presents the chemical composition and the BET surface area of the as-is ion-adsorption clay sample and each size fraction. As shown, the total REEs (TREE) content is 3,204 mg/kg or 0.32 wt%. It can also be seen that the REE content decreases with increasing particle size. The same trend is observed for all other elements except for silicon that has an opposite trend. In terms of size fraction weigh percentages, S1 (< 0.25 mm) accounts for 48% of the total mass, followed by S2 (0.25–0.5) 15% and then S3 (0.5–2 mm) that accounts for 37% of the total mass. Based on the compositional analysis, Y, Ce, and La are the major REEs in this ion-adsorption clay sample. It also contains high amount of Nd, but it also contains good amount of Dy (which is a critical HREE), which makes this ionic clay a valuable source of HREEs.

**Table 1:** Elemental composition of the ion-adsorption clay and its size fractions: S1 (< 0.25 mm), S2 (0.25–0.5), S3 (0.5–2 mm).

Sample Name	Ionic clay—as-is	S1	S2	S3
Particle Size	–	< 0.25 mm	0.25–0.5 mm	0.5–2 mm
BET Surface area (m^2^/g)	30.8	32.5	24.7	24.9
Mass fraction (%)	100	48	15	37
Element	ppm (mg/kg)	ppm	ppm	ppm
Sc	28.8	30.3	21.1	22.7
Y	898.5	1262.4	887.6	431.6
La	464.7	699.6	361.6	210.2
Ce	816.0	1139.2	656.7	496.5
Pr	100.5	154.8	77.0	46.8
Nd	423.2	644.4	334.1	196.1
Sm	68.9	104.5	56.0	32.9
Eu	2.3	3.4	2.0	1.0
Gd	78.9	111.4	68.6	37.6
Tb	10.7	14.7	9.3	5.2
Dy	120.9	173.7	118.5	64.5
Ho	24.7	34.7	25.1	13.2
Er	79.6	112.5	81.8	43.6
Tm	10.6	14.9	10.8	6.0
Yb	63.0	87.6	63.0	38.4
Lu	13.0	15.8	11.9	6.9
Total REEs	3204.0	4603.9	2785.3	1653.4
Th	152.1	234.7	118.7	83.0
U	1.6	2.4	1.3	1.0
	wt%	wt%	wt%	wt%
Fe	8.3	9.2	7.6	6.9
Al	8.4	12.5	6.8	3.4
Si	31.5	24.0	35.1	40.4
P	0.2	0.2	0.2	0.2

In terms of bulk elements, the ion-adsorption clay contains high amount of Fe (8.3 wt% which is almost the same as the Al content (8.4 wt%)). The presence of Al indicates the presence of clay minerals such as kaolinite, halloysite, or other clay minerals as well as other minerals containing Al. The presence of high amount of Fe indicates that the ion-adsorption clay is rich in other minerals beside the clay minerals. This indicates a major difference between this ion-adsorption clay deposit sample and the ones from southern China which mainly contain clay minerals and very low amount of Fe^22–26^.

In terms of surface area, S1 (< 0.25 mm) has the largest surface area of 32.5 m^2^/g followed by S2 (0.25–0.5 mm) and S3 (0.5–2 mm) which have very similar surface areas (24.7 and 24.9 m^2^/g). The nonlinear relationship between the surface area and the total amount of REEs indicates that higher amount of REEs within the smaller size fractions is not simply a function of the surface area of the material.

**Figure 1:**
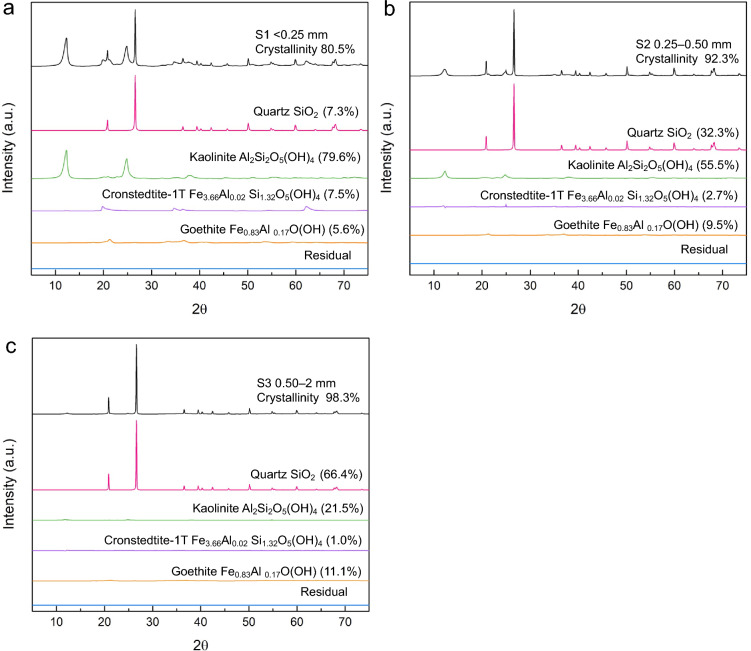
XRD diffractograms and Rietveld analysis of the ion-adsorption clay size fractions: S1 (< 0.25 mm), S2 (0.25–0.5), S3 (0.5–2 mm).

The mineralogy of the ionic clay fractions (S1–S3) were determined by X-ray diffraction and Rietveld analysis was used to quantify the amount of each mineral. More details about these methods are provided in the Experimental section. The XRD diffractograms are presented in Fig. 1a-c. The clay mineral was found to be kaolinite (Al_2_Si_2_O_5_(OH)_4_), and other minerals include quartz (SiO_2_), Cronstedtite-1T (Fe_3.66_Al_0.02_ Si_1.32_O_5_(OH)_4_), and goethite (Fe_0.83_Al _0.17_O(OH)). The goethite in the XRD diffractograms have Fe substitution by Al which is a natural occurrence reported in the literature^27^. The elemental composition in Table 1 shows that with increasing particle size, the Al content decreases and the Si content increases; this trend is consistent with the mineral phase composition presented in Fig. 1a-c as larger size fraction contains more quartz and less kaolinite. The detection of P (Table 1) can be an indication of monazite or xenotime that contains mineralized REEs, but because of its low content, it was not detected by XRD.

Table 2 summarizes the Rietveld analysis results along with the composition of select elements from Table 1. The smallest size fraction S1 (< 0.25 mm) has the highest content of TREEs (4604 ppm), which is associated with its highest content of clay minerals (kaolinite, 79.6 wt%) as shown in Fig. 1a. The REE content decreases with increasing the particle size in line with the decrease of kaolinite content with increasing particle size. It is obvious that REEs in this ion-adsorption clay sample are associated with kaolinite (the clays mineral) as adsorbed ions, and later, the amount of desorbable REEs is quantified in this study.

**Table 2:** Rietveld analysis results indicating the mineral phase quantification along with the composition of Al, Fe, Si, and TREE in each ion-adsorption clay size fraction: S1 (< 0.25 mm), S2 (0.25–0.5), S3 (0.5–2 mm).

Samples	Kaolinite (Al_2_Si_2_O_5_(OH)_4_) (%)	Quartz (SiO_2_) (%)	Cronstedtite-1T (Fe_3.66_Al_0.02_ Si_1.32_O_5_(OH)_4_) (%)	Goethite (Fe_0.83_Al_0.17_O(OH) (%)	Al (wt%)	Fe (wt%)	Si (wt%)	TREE (mg/kg, ppm)
S1	79.6	7.3	7.5	5.6	12.5	9.2	24.0	4603.9
S2	55.5	32.3	2.7	9.5	6.8	7.6	35.1	2785.3
S3	21.5	66.4	1.0	11.1	3.4	6.9	40.4	1653.4

**Figure 2:**
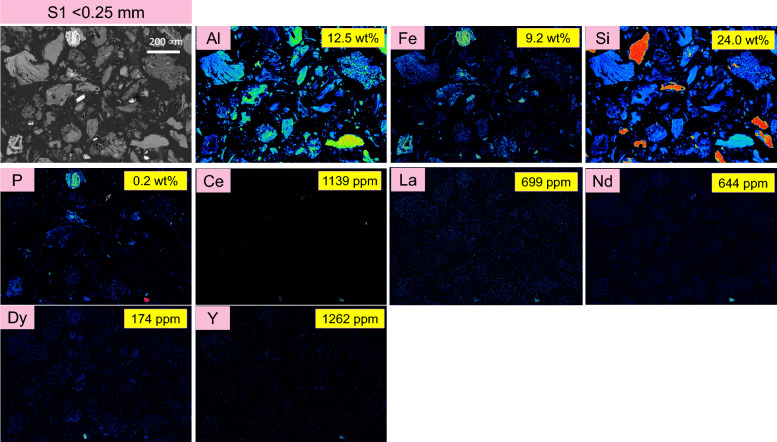
EPMA back scattered electron image and elemental mapping results for the target elements: Al, Fe, Si, P, Ce, La, Nd, Y, and Dy in S1 (< 0.25 mm) size fraction of the ion-adsorption clay. For comparison, the compositional analysis obtained from ICP-OES and ICP-MS are presented in yellow boxes on each panel.

**Figure 3:**
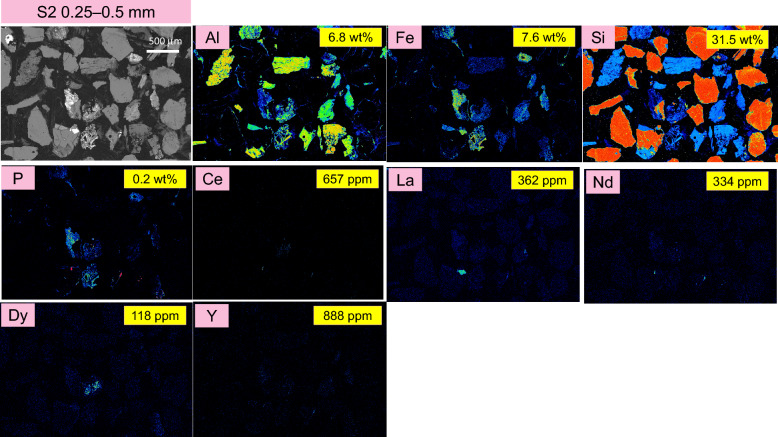
EPMA back scattered electron image and elemental mapping results for the target elements: Al, Fe, Si, P, Ce, La, Nd, Y, and Dy in S2 (0.25–0.5 mm) size fraction of the ion-adsorption clay. For comparison, the compositional analysis obtained from ICP-OES and ICP-MS are presented in yellow boxes on each panel.

**Figure 4:**
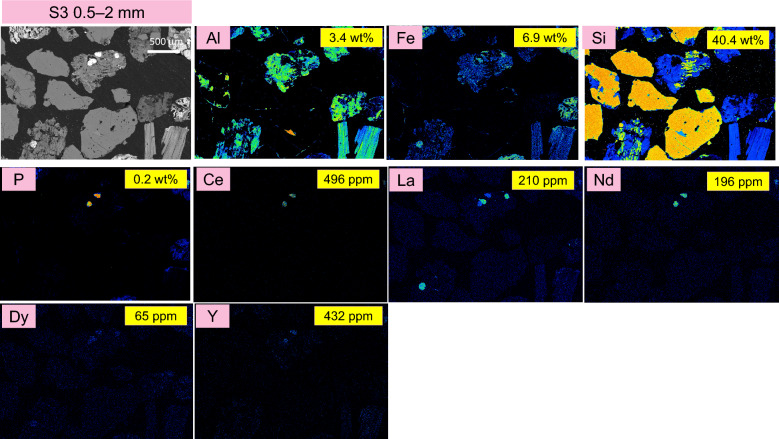
EPMA back scattered electron image and elemental mapping results for the target elements: Al, Fe, Si, P, Ce, La, Nd, Y, and Dy in S3 (0.5–2 mm) size fraction of the ion-adsorption clay. For comparison, the compositional analysis obtained from ICP-OES and ICP-MS are presented in yellow boxes on each panel.

Figures 2, 3 and 4 display the results obtained from Electron Probe Microanalysis (EPMA), including backscattered electron (BSE) images and elemental mapping for the three different size fractions of the ion-adsorption clay sample (S1 to S3), respectively. Additionally, for comparison, the elemental composition of the analyzed elements as measured by ICP-OES and ICP-MS is presented in yellow boxes.

Observations from the BSE images reveal that the particle size increases from S1 to S3. There are significant decreases in Al content, while the Fe content decreases to some extent from S1 to S3. Conversely, the Si content increases from S1 to S3, which is consistent with the increasing presence of SiO_2_ minerals in the samples as shown in the XRD diffractograms in Fig. 1.

The detected presence of Nd, La and Ce (as proxies for LREEs) in the EPMA elemental maps is attributed to possibly monazite mineral, as they are co-located with phosphorus (P). It is important to note that EPMA could not detect the ion adsorbed REEs on the surface of kaolinite. The values for La, Nd and Ce presented in the yellow boxes represent the total La, Nd, and Ce, originating from both the ion adsorbed REEs and mineralized forms in possibly monazite. The elemental mapping of Dy and Y (as proxies for HREEs) was also detected. The results indicate that Y is not present in these samples, confirming that no xenotime is present in this ion-adsorption clay sample.

Since the concentration of ion-adsorbed REEs decreases with increasing particle size from S1 to S3, the values presented in the yellow boxes also show a decreasing trend from S1 to S3.

**Figure 5:**
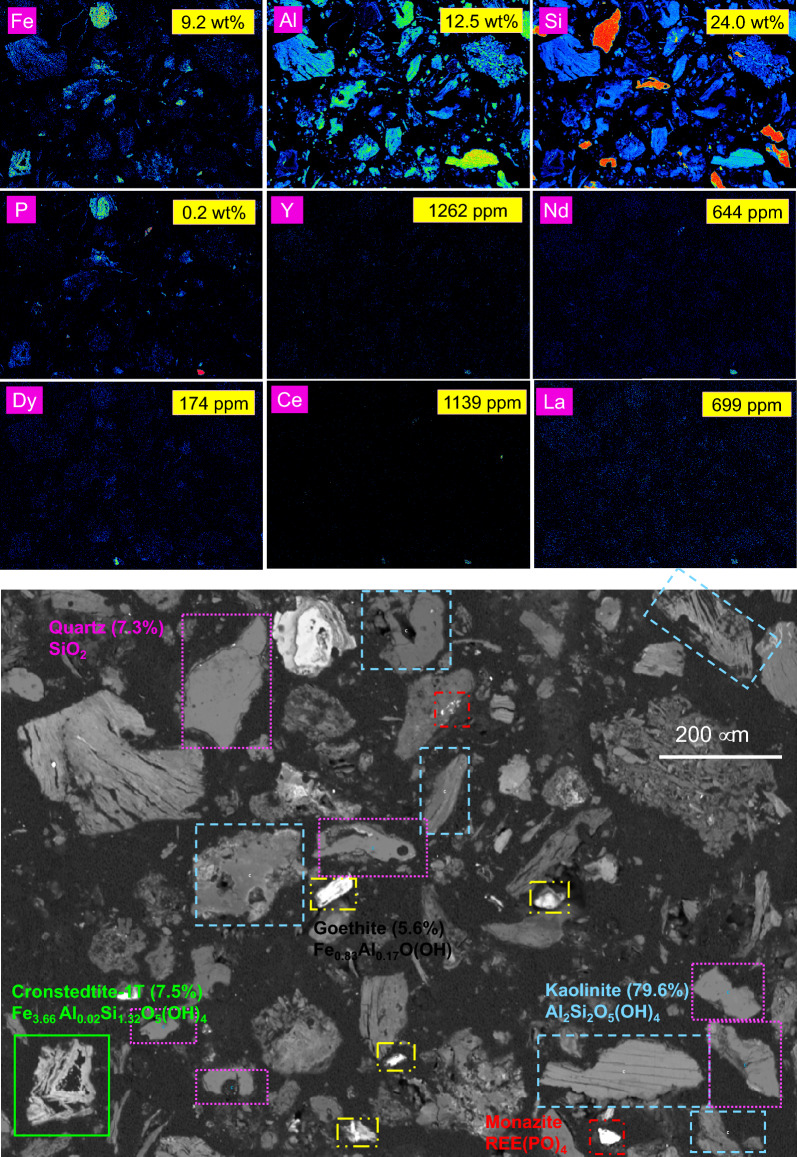
EPMA back scattered electron image along with mineral phase identification and Rietveld analysis results. Elemental mapping results for the target elements: Al, Fe, Si, P, Dy, Y, La, Ce, and Nd in S1 ion-adsorption clay size fraction (< 0.25 mm) are also presented. For comparison, the compositional analysis obtained from ICP-OES and ICP-MS are presented in yellow boxes on each panel.

Figure 5 provides a comprehensive overview of the phase identification of different minerals in the S1 clay size fraction, utilizing elemental mapping. For comparison, the figure also includes results from Rietveld analysis and compositional analysis. This multifaceted figure offers valuable insights into the distribution of various minerals within the ion-adsorption clay sample, incorporating elemental composition, Rietveld analysis of mineral phases, particle morphology, mineral distribution, and elemental mapping, all within a single image. Notably, the fraction with the smallest particles (S1) contains the highest concentration of kaolinite, which aligns with the observed higher content of TREEs in this specific fraction.

### Desorption of desorbable REEs

**Figure 6:**
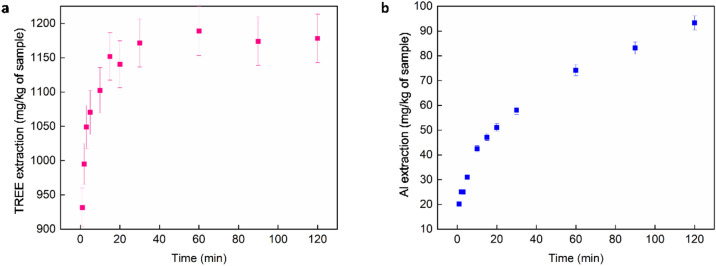
Desorption Kinetics for: a) TREE and b) Al using 0.15 mol/L ammonium sulfate, L/S of 3 mL/g, at pH 3 and ambient temperature. The error bars represent three replicates.

To determine the equilibrium time, desorption kinetic experiments were performed with 0.15 mol/L (NH_4_)_2_SO_4_, L/S of 3 mL/g at pH 3. These conditions have shown to results in maximum extraction of desorbable REEs^28^. As shown in Fig. 6a, the reaction kinetics is very fast and TREE extraction reaches equilibrium within 30 min. On the contrary to REEs, extraction of Al was 58 $\frac{{mg}_{Al}}{{kg}_{sample}}$ at 30 min and 92 $\frac{{mg}_{Al}}{{kg}_{sample}}$ at 120 min, which was low and indicated that the studied conditions cannot extract mineralized bulk elements; however, very small amount of Al can be desorbed from the clay mineral, consistent with a previous report in the literature ^28^. It is also observed that Al extraction does not reach equilibrium and it keeps increasing with increasing time; therefore, it is desirable to keep the desorption time as small as possible, to minimize the extraction of Al (Fig. 6b). Extraction of Fe was very low, below 5 $\frac{{mg}_{Fe}}{{kg}_{sample}}$ which indicates there are no desorbable Fe in the ion-adsorption clay sample and mineralized Fe cannot be extracted under the mild conditions used for extraction process.

To determine the distribution of different REE adsorption forms (physiosorbed and chemisorbed) within the as-is ion-adsorption clay sample and its three size fractions (S1–S3), two sets of tests were conducted. One set involved the presence of 0.15 mol/L (NH_4_)_2_SO_4_, while the other did not include (NH_4_)_2_SO_4_. In both sets of experiments, sulfuric acid was added to the slurry containing the clay and lixiviant, reducing the slurry's pH to 3. The same experiments were repeated at the clay's native pH (~ 5) without the addition of sulfuric acid. By comparing the desorption efficiencies $\frac{{mg}_{REE}}{{kg}_{sample}}$ under these two conditions, the contributions from the different REE adsorption forms were calculated. The calculation method is shown in the Experimental section.

**Figure 7:**
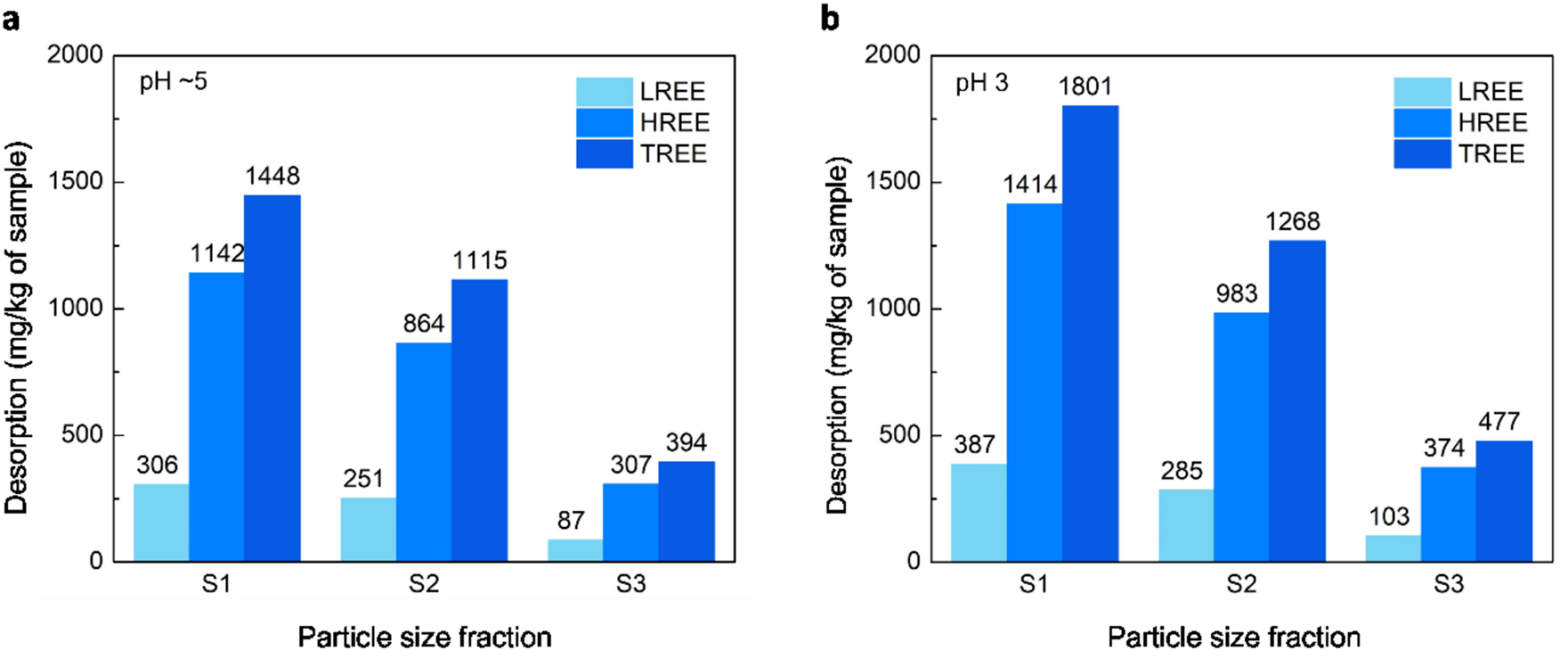
Desorption in $\frac{{mg}_{REE}}{{kg}_{sample}}$ for the three size fractions S1 (< 0.25 mm), S2 (0.25–0.5), S3 (0.5–2 mm) using 0.15 mol/L ammonium sulfate at L/S of 3 mL/g and two pH: a) native pH (~ 5), b) pH 3.

Figure 7 illustrates the desorption results for the three size fractions (S1–S3) at two different pH levels: the native (~ 5) pH and pH 3. Desorption results at the native pH (~ 5) are associated with physiosorbed REEs, while those at pH 3 are linked to chemisorbed REEs.

Figure 7a demonstrates that smaller particles contain more physiosorbed REEs compared with larger particles, which aligns with the higher kaolinite content in smaller particles. Additionally, it is observed that more heavy rare earth elements (HREEs) are desorbed compared with light rare earth elements (LREEs) despite the fact that the concentration of LREEs is higher than HREEs in this sample. The reason is that most of the LREEs in this sample are in the mineralized form and cannot be desorbed under the mild conditions of the desorption process and they require high temperature and highly acidic conditions to be extracted. On the contrary, the HREEs are adsorbed on the ion-adsorbed clay and they can be desorbed during the desorption process. Table 3 presents the occurrence modes of LREEs and HREEs in each size fraction which confirms LREEs are mostly in the mineralized form and HREEs are mostly physiosorbed and chemisorbed.

**Table 3:** Distribution of REE occurrence modes of LREEs and HREEs in the three size fractions S1 (< 0.25 mm), S2 (0.25–0.5), S3 (0.5–2 mm).

Sample	Content mg/kg		Physically adsorbed (%)		Chemically adsorbed (%)		Mineralized (%)	
	LREE	HREE	LREE	HREE	LREE	HREE	LREE	HREE
S1	2887.6	1716.3	11%	67%	3%	16%	86%	17%
S2	1577.1	1208.0	16%	72%	2%	10%	82%	18%
S3	1043.8	609.4	8%	50%	2%	11%	90%	39%

Based on the compositional data presented in Table 1, the percentage of LREEs and HREEs in the as-is clay, as well as S1–S3 size fractions, are 63% and 38%, respectively (except for S2, which is 57% LREEs and 43% HREEs). According to the desorption data in Fig. 7a,b, the desorbed amount of LREEs in S1–S3 is 22%, while that of HREEs is 78%. This result suggests that different particle size fractions exhibit a similar preference for adsorbing both LREEs and HREEs.

Table 3 highlights the differences in occurrence modes between light rare earth elements (LREEs) and heavy rare earth elements (HREEs). It indicates that while LREEs are more abundant, they are predominantly mineralized and require more intensive conditions for extraction. In contrast, most HREEs are adsorbed on the surface. Additionally, both LREEs and HREEs tend to favor physical adsorption over chemisorption.

Table 4 provides an overview of the TREE content in each size fraction and the percentage of physiosorbed, chemisorbed, and mineralized REEs within each size fraction. It is evident that this ion-adsorption clay contains a significant amount of mineralized REEs that cannot be desorbed under typical conditions and necessitate more aggressive treatments such as acid baking followed by water leaching for extraction. Moreover, the table highlights that the majority of the desorbable REEs in this clay are physiosorbed, accounting for over 80% of the total. The chemisorbed REEs can be adsorbed onto the clay minerals like kaolinite, as well as associated minerals such as quartz and goethite. Further investigation into this chemisorption mechanism will be explored in a future study.

**Table 4:** Distribution of REE occurrence modes in the three size fractions S1 (< 0.25 mm), S2 (0.25–0.5), S3 (0.5–2 mm).

Sample	TREE content	TREE: Physically adsorbed	TREE: Chemically adsorbed	TREE: Desorbable	TREE: Mineralized
	mg/kg	(%)	(%)	(%)	(%)
S1	4603.9	31	8	39	61
S2	2785.3	40	5	45	55
S3	1653.4	24	5	29	71

### Comparison between the studied ion-adsorption clay and those from other regions

The ion-adsorption clay in this study differs from traditional ones in a few respects, including mineral compositions and REE occurrence modes. Traditional ion-adsorption deposits typically contain less than 2 wt% iron^22–26^. However, in this South American sample, two primary iron minerals, Cronstedtite-1T and goethite, are identified, contributing 8.3% of the sample's weight in iron. These minerals are associated with 20.5%, 11.1%, and 17.2% of chemisorbed REEs in size fractions S1, S2, and S3, respectively, suggesting that using conventional desorption methods may lead to significant REE losses. Consequently, it is essential to reduce the pH from native pH to 3 to efficiently desorb REEs from this deposit. Adjusting pH to 3 could enhance REE recovery by 25.8%, 12.5%, and 20.8% for each respective size fraction compared with native pH.

Additionally, monazite, identified as the possible mineralized form of REEs and not reported in conventional deposits, requires aggressive extraction conditions, such as treatment with 98 wt% H_2_SO_4_ or 70 wt% NaOH. In this South American ion-adsorption clay, over half of the REEs are mineralized, predominantly as LREEs. Given the co-existence of adsorbed and mineralized REEs, and the marked tendency for LREEs to be mineralized and HREEs to be adsorbed, a two-step recovery process can be utilized if 100% recovery of all REEs is targeted: 1) Desorption to extract most HREEs, and 2) Intense leaching to recover most LREEs.

## Conclusions

This research aimed to provide a comprehensive understanding of the complex interplay between particle size, surface area, morphology, mineralogical composition, elemental mapping, and REE adsorption forms in an ion-adsorption clay sample sourced from South America. This ion-adsorption clay sample stands out from the majority of previously studied ion-adsorption clay samples as it contains a different combination of REE adsorption forms, including chemisorption, which is not as prevalent in other samples primarily characterized by physisorption.

The study involved the fractionation of the ion-adsorption clay sample into three size categories: S1 (< 0.25 mm), S2 (0.25–0.5 mm), and S3 (0.5–2 mm). Each fraction underwent an extensive characterization process to determine its composition, mineralogical content, surface area, morphological features, elemental mapping, and the forms responsible for REE incorporation. The following key discoveries were drawn from this investigation:

1. Particle Size, mineral compositions, and REE quantification: Smaller particles (S1) exhibited a higher proportion of REEs compared with larger particles (S3). This observation aligns with the greater presence of kaolinite, a mineral conducive to adsorbed REEs, in the smaller particles. While S2 has a similar specific surface area as S3 and much less than S1, it has the highest percentage of adsorbed REEs. This discovery indicates that the desorption/adsorption modes is not only affected by the particle sizes and surface area, but also the mineral composition.
2. Adsorption forms: The majority of desorbable REEs were found to be physiosorbed, accounting for over 80% of the total REEs. Chemisorbed REEs, on the other hand, could be associated with various minerals, including kaolinite, quartz, and goethite.
3. Mineralized REEs: The ion-adsorption clay sample contained a substantial amount of mineralized REEs that were not readily desorbed. The mineralized REEs are mostly contributed by the LREEs. These mineralized REEs necessitate more rigorous extraction methods, such as acid baking followed by water leaching.
4. Distribution of LREEs and HREEs: The study revealed that although the ion-adsorption clay contained higher concentration of LREEs than HREEs, they were mostly in the mineralized form that cannot be desorbed under the mild desorption condition. On the contrary, the HREEs were mostly in the form of physiosorbed and then chemisorbed species; therefore, they could be easily desorbed during the desorption process.
5. HREEs were preferentially adsorbed in higher quantities than LREEs. Most LREEs are mineralized in the sample while most of HREEs are adsorbed on the surface.

In summary, this study has shed light on the intricate relationship between particle size, mineralogical composition, surface characteristics, and REE adsorption forms within an atypical ion-adsorption clay sample. These findings contribute to a deeper understanding of the factors influencing REE adsorption in ion-adsorption clays and highlight the importance of considering different adsorption forms in future extraction processes.

It is important to mention that this manuscript is based on the analysis of a single sample. Future research will aim to investigate additional samples from various drillholes in the same region to broaden the scope and enhance the generalizability of the study's findings.

## Methodology

### Materials and reagents

The ion-adsorption clay sample was obtained from an undisclosed source in South America. Nitric acid (ACS Reagent Grade, 68.0–70.0 wt% Assay, VWR), sulfuric acid (ACS Reagent Grade, 95.0–98.0 wt% Assay, VWR), ammonium sulfate (ACS Reagent Grade, > 99.0 wt%, VWR), and sodium hydroxide (ACS Reagent Grade, 50.0 wt% Assay, VWR) were used to perform characterization and experiments. Fusion flux (ultrapure, Li_2_B_4_O_7_/LiBO_2_/LiBr 49.75 wt%/49.75 wt%/0.50 wt%, SCP Science) was used for alkali fusion. Deionized water (resistivity of 18.2 MΩ-cm) produced by the Milli-Q Integral water purification system of MilliporeSigma (Merck KGaA, Darmstadt, Germany) was used for making the solutions. Certified multi-element standard stock solutions (Inorganic Ventures) were used for calibration of analytical instruments.

### Sample characterization

#### Sample preparation

The samples were first fully dried and then finely pulverized using a Mixer Mill 400 for a duration of 3 min at a frequency of 25 Hz. This ensured that the samples were homogenized and suitable for subsequent analyses.

#### Compositional characterization

Compositional analysis was conducted using borate (alkali) fusion. A fusion fluxer known as Claisse LeNeo was used for this purpose. The resulting compositions were analyzed for REEs, thorium (Th), uranium (U), and bulk elements. Inductively coupled plasma mass spectrometry (ICP-MS) with a PerkinElmer NexION 2000 instrument was utilized to measure the concentrations of REEs, Th, and U. Bulk element concentrations were determined using inductively coupled plasma optical emission spectrometry (ICP-OES) with a PerkinElmer Optima 8000 instrument.

#### Mineralogical analysis

The pulverized samples were placed in shallow well sample holders and analyzed using a Rigaku MiniFlex 600 Diffractometer for X-ray diffraction (XRD). The analysis spanned a 2θ range from 5° to 75° with a scanning rate of 1.25°/min. Rietveld analysis, performed using the integrated X-ray powder diffraction software (PDXL). Rietveld analysis adjusts a theoretical diffraction pattern to match the experimental data by refining a structural model of the material. It involves modifying various parameters, including lattice constants, atomic positions, and thermal vibrations, to minimize the difference between the observed and calculated diffraction patterns. This is quantitatively assessed using a statistical parameter, often the chi-squared (χ^2^) value lower than 5. Details of the database are provided in Table 5. The analysis revealed that the phases of cronstedtite-1T and goethite remained consistent across the three samples, while the amounts of kaolinite and quartz varied with particle size.

**Table 5:** The DB card number for each phase.

Phase name	Formula	DB card number
S1		
Kaolinite	(Al_2_Si_2_O_5_(OH)_4_)	04-013-2815
Quartz	(SiO_2_)	04-005-4718
Cronstedtite-1T	(Fe_3.66_Al_0.02_ Si_1.32_O_5_(OH)_4_)	04-013-9159
Goethite	(Fe_0.83_Al_0.17_O(OH)	04-013-6663
S2		
Kaolinite	(Al_2_Si_2_O_5_(OH)_4_)	01-083-4643
Quartz	(SiO_2_)	04-005-4718
Cronstedtite-1T	(Fe_3.66_Al_0.02_ Si_1.32_O_5_(OH)_4_)	04-013-9159
Goethite	(Fe_0.83_Al_0.17_O(OH)	04-013-6663
S3		
Kaolinite	(Al_2_Si_2_O_5_(OH)_4_)	9,009,234
Quartz	(SiO_2_)	1,011,172
Cronstedtite-1T	(Fe_3.66_Al_0.02_ Si_1.32_O_5_(OH)_4_)	04-013-9159
Goethite	(Fe_0.83_Al_0.17_O(OH)	04-013-6663

### Morphological and elemental mapping analysis

To understand the morphology of the samples and map the distribution of elements within them, an electron probe microanalyzer (EPMA) with a JEOL JXA8230 5-WDS Electron Microprobe was utilized. The operating conditions used in the EPMA analysis are presented in Table 6. For EPMA analysis, the samples without pulverization were mixed with epoxy-forming pellets and subsequently polished to create cross-sections for analysis.

**Table 6:** Operating conditions used in EPMA.

Parameters	Value
Accelerating voltage	10.0 kV
Probe current	30 nA
Mode	SPOT
Probe diameter (nom)	1 µm
Dwell time	10.00 ms
Direction	Single
Points	750 $\times$ 535
Interval	X: 2.00 Y:2.00

### Surface area analysis

The surface area of the samples was analyzed using a NOVA 1200e BET nitrogen absorption instrument. Each sample was individually subjected to degassing for 7 h at a temperature of 200 °C under vacuum conditions. After the degassing process, nitrogen (N_2_) gas physisorption analysis was performed. This analysis used the static volumetric method, which measures the amount of N_2_ gas adsorbed by the sample at different relative pressures. The surface area was calculated using the Brunauer–Emmett–Teller (BET) method. This method involves creating a linear plot of the quantity $\frac{1}{W(\left(\frac{{P}_{0}}{P}\right)-1)}$ versus $\frac{{P}_{0}}{P}$, where W represents the mass of the analyte powder, P_0_ is the vapor pressure of the adsorbate gas (N_2_ in this case), and P is the relative pressure of the adsorbate gas. It is important to note that the BET method typically applies to a limited region of the adsorption isotherm. For most solids, especially when using N_2_ as the adsorbate, this range falls within the $\frac{{P}_{0}}{P}$ range of 0.05 to 0.3. The specific surface area was determined based on the BET plot, considering factors such as the sample surface, the cross-sectional area of the adsorbate gas (which is 16.200 Å^2^ for N_2_), and the mass of the analyte powder.

### Experimental procedure

#### Wet sieving

Wet sieving was performed by sieving the as-is ion-adsorption clay with three sieves (mesh 10, mesh 35, and mesh 60, Science First) to separate particles into 4 sizes: < 0.25 mm (S1), 0.25–0.5 mm (S2), 0.5–2 mm (S3), and > 2 mm. The particles larger than 2 mm were not investigated. To avoid the agglomeration of fine particles to the large particles, samples on each sieve were washed with deionized water. The smallest particles (S1) were washed off and collected in the bottom tray which were then separated using vacuum filtration to collect solids. All samples were dried at room temperature for further experiments. The wash water was analyzed with ICP-MS and no REE losses were detected which confirms the appropriateness of the methodology.

#### Desorption procedure

A 5-g sample of the clay was analyzed using a moisture analyzer (Torbal AST120) to determine its moisture content. Next, 50 g of the clay sample was mixed with water and 0.15 mol/L ammonium sulfate ((NH_4_)_2_SO_4_) at a liquid to solid (L/S of 3 mL/g) and a controlled pH (native (~ 5) or 3). The pH was maintained within a range of ± 0.1 of the setpoint through periodic additions of a 20 wt% sulfuric acid solution. To determine the L/S ratio, the initial humidity of the clay was taken into account, and the amount of liquid added was calculated based on the mass of the dry clay. Kinetic investigations determined that a residence time of 30 min was sufficient for all experiments. The agitation rate during this time was set at 200 rpm. After completing the experiment, the slurry was vacuum filtered using Whatman Grade 3 filter paper. A 5-g sample of the wet filter cake was taken and analyzed to measure its humidity. The filtrate obtained after desorption was sampled using a syringe and nylon syringe filters (Basix, 0.45 µm) were attached to the syringe to ensure that any solids were filtered out as the samples were extracted. The sample was then diluted with a 5 wt% nitric acid (HNO_3_) solution for stabilization. The concentration of REEs and bulk elements was measured using ICP-MS and ICP-OES, respectively. Following the desorption experiments, the solid filter cake was washed three times with 100 mL of deionized water to remove any residual REEs and ammonium ions from the surfaces of the solid particles. After washing, the filter cake was dried in an oven at 50 °C for 24 h.

The desorption efficiency was calculated as the mg of the element extracted from 1 kg of the ion-adsorption clay which was calculated using mass balance with the following parameters: m_H2O_ = mass of added water, m_clay0_ = mass of dry clay entering the process, m_hum0_ = mass of water in the clay, mass_ammonium sulfate_ = mass of ammonium sulfate used for desorption, m_H2SO4_ = mass of sulfuric acid, m_sol_ = mass of recovered filtrate, and m_hum1_ = mass of water in the filter cake. Using mass balance Eq. (6), m_sol_ (mass of recovered filtrate) can be calculated (it is assumed that there are no mass losses):

6 $${m}_{clay0}+{m}_{hum0}+{m}_{H2O}+{m}_{ammonium sulfate}+{m}_{H2SO4}={m}_{clay0}+{m}_{sol}+{m}_{hum,f}$$

7 $${{m}_{sol}=m}_{clay0}+{m}_{hum0}+{m}_{H2O}+{m}_{ammonium sulfate}+{m}_{H2SO4}-{m}_{clay0}-{m}_{hum,f}$$

8 $$Desorbed amount \left(\frac{mg}{{kg}_{clay}}\right)=\frac{{c}_{i,sol}\times {V}_{sol}\times 1000}{{m}_{clay,0}}$$

where c_i,sol_ is the measured concentration of element i in the solution after desorption (mg/L), V_sol_ (L) is the volume of the solution which is calculate using m_sol_ (g) from Eq. (7) and measured density of the solution (g/mL), and m_clay,0_ is mass of the dry clay entering the process (g).

#### Determination of REE incorporation mechanism

For determining the percentage of each REE incorporation mechanism (physiosorbed and chemisorbed) within the as-is ion-adsorption clay and its three fractions (S1–S3), two types of experiments were performed, one in the presence of 0.15 mol/L (NH_4_)_2_SO_4_ and one without (NH_4_)_2_SO_4_. The slurry containing the clay and lixiviant in both tests was subjected to sulfuric acid addition and the pH of the slurry was decreased to 3. The same experiments were repeated at the native pH (~ 5) without adding sulfuric acid. By comparing the desorption efficiencies (mg_REE_/kg_sample_) at the two conditions, contributions from the different REEs (physiosorbed and chemisorbed) were calculated. The initial concentration of REEs in the starting solution (water + ion-adsorption clay) corresponds to the amount of water soluble REEs in the ion-adsorption clay, which is almost zero. The proportion of physically adsorbed REEs was determined as the difference in extracted concentrations between the starting solution (pH ~ 5) and the solution with added ammonium sulfate but no pH adjustment (pH 4.5), as given in Eq. (9). The chemically adsorbed fraction was determined as the difference in extracted concentrations between the solution with only ammonium sulfate (pH 4.5) and pH 3 (which is the point at which mineral dissolution starts, determined in the previous study^28^), Eq. (10). The desorbable fraction is the sum of the physically and chemically adsorbed fractions. The mineralized fraction was determined as the difference in extracted concentrations between the onset of mineral dissolution (pH 3) and the total REE content of the clay (Eq. (11)). It should be noted that although dissolution of the mineral phases begins at pH < 3, this dissolution accounts for a small amount of the total REEs in the clay, which require highly acidic conditions at elevated temperature to fully dissolve REEs (for example acid baking − water leaching).

9 $$Physiosorbed \left(\%\right)=\frac{\left({TREE}_{{desor,\left(N{H}_{4}\right)}_{2}S{O}_{4}}-{TREE}_{Nat pH}\right)}{{TREE}_{clay,0}}\times 100\%$$

10 $$Chemisorbed \left(\%\right)=\frac{\left({TREE}_{desor,pH 3}-{TREE}_{{desor,\left(N{H}_{4}\right)}_{2}S{O}_{4}}\right)}{{TREE}_{clay,0}}\times 100\%$$

11 $$Mineralized \left(\%\right)=\frac{\left({TREE}_{clay,0}-{TREE}_{desor,pH 3}\right)}{{TREE}_{clay,0}}\times 100\%$$

where ${TREE}_{{desor,\left(N{H}_{4}\right)}_{2}S{O}_{4}}$ is the TREE (total REEs) desorption efficiencies (mg_REE_/kg_sample_) at the native pH (~ 5) with 0.15 mol/L (NH_4_)_2_SO_4_, ${TREE}_{Nat pH}$ is the TREE desorption efficiencies (mg_REE_/kg_sample_) at the native pH (~ 5) without (NH_4_)_2_SO_4_, ${TREE}_{desor,pH 3}$ is the TREE desorption efficiencies (mg_REE_/kg_sample_) at pH 3, and ${TREE}_{clay,0}$ is TREE concentration (mg/kg) in the ion-adsorption clay sample.

## Data Availability

The data will be made available upon request. Please contact the corresponding author, Dr. Gisele Azimi (g.azimi@utoronto.ca).
